# Medication related problems in elderly patients treated with antipsychotics

**DOI:** 10.1192/j.eurpsy.2024.103

**Published:** 2024-08-27

**Authors:** M. Stuhec

**Affiliations:** Clinical Pharmacology, Medical Faculty, Maribor University & Ormoz Psychiatric Hospital, Maribor, Slovenia

## Abstract

**Abstract:**

Elderly patients treated with antipsychotics are often prescribed multiple medications simultaneously (polypharmacy), leading to an increased risk of various medication-related problems, including irrational polypharmacy, drug-drug interactions, and potentially inappropriate medications (PIMs). Polypharmacy may be rational with clear indications and benefits, but it becomes irrational when better alternatives are available. This irrational polypharmacy is associated with poorer clinical and economic outcomes, including a higher mortality rate. Antipsychotic polypharmacy in elderly patients with schizophrenia is not well-studied and, therefore, is not generally recommended. Long-term antipsychotic use in patients with dementia has also been linked to a higher mortality rate. PIMs, representing a greater risk than benefit, are prevalent in mental health institutions at all healthcare levels, with a prevalence ranging from 40% to 60%. Antipsychotics, along with benzodiazepines, are among the most commonly included in PIMs and polypharmacy in these institutions. Moreover, antipsychotics are frequently implicated in potential severe drug-drug interactions in elderly patients with mental disorders, particularly with antibiotics and antiarrhythmics.

Unfortunately, the existing treatment guidelines and meta-analyses mostly do not cover these aspects, which represent a gap between the ‘real clinical’ pharmacology and treatment guidelines. The speaker will summarize the medication-related problems in this population and present practical recommendations for daily clinical practice.

**Disclosure of Interest:**

None Declared

